# High-dose carbon-ion based radiotherapy of primary and recurrent sacrococcygeal chordomas: long-term clinical results of a single particle therapy center

**DOI:** 10.1186/s13014-020-01647-8

**Published:** 2020-08-24

**Authors:** Tilman Bostel, Matthias Mattke, Nils Henrik Nicolay, Thomas Welzel, Daniel Wollschläger, Sati Akbaba, Arnulf Mayer, Tanja Sprave, Jürgen Debus, Matthias Uhl

**Affiliations:** 1 Department of Radiation Oncology, University Medical Center Mainz, Langenbeckstrasse 1, 55131 Mainz, Germany; 2 Clinical Cooperation Unit Radiation Oncology, German Cancer Research Center (DKFZ), Im Neuenheimer Feld 280, 69120 Heidelberg, Germany; 3 Department of Radiation Oncology, University Hospital of Heidelberg, Im Neuenheimer Feld 400, 69120 Heidelberg, Germany; 4 Department of Radiation Oncology, University Hospital of Freiburg, Robert-Koch-Strasse 3, 79106 Freiburg, Germany; 5 Institute of Medical Biostatistics, Epidemiology and Informatics (IMBEI), University Medical Center Mainz, Mainz, Germany

## Abstract

**Background:**

This study aimed to analyze the oncological long-term results and late toxicity of carbon ion-based radiotherapy (RT) of patients with sacral chordoma and to identify potential prognostic factors for local control (LC) and overall survival (OS).

**Methods:**

A total of 68 patients with sacral chordoma treated at the Heidelberg Ion Beam Therapy Center were included in this study. Of these 52 patients (77%) received a primary RT and 16 patients (23%) received a RT in a recurrent situation. All patients were treated with carbon ion RT (CIRT), either in combination with photons (*n* = 22; 32%) or as a monotherapy (*n* = 46; 68%), with a median radiation dose of 66 Gy RBE (range 60–74 Gy). In 40 patients (59%), RT was performed in the postoperative situation. Postoperative care included regular MRI scans. Local progression was defined as an enlargement of the maximum tumor diameter by 10% or a new tumor growth within the planning target volume (PTV). LC and OS were determined using the Kaplan-Meier method. Furthermore, the relevance of various prognostic factors for LC and OS was assessed by univariate and multivariate analysis.

**Results:**

The median follow-up period was 60 months (range 1.3–97.4 months). The 5-year rates for LC, progression-free survival, metastasis-free survival and OS were 53, 53, 52 and 74%, respectively. Local recurrence was observed in 31 patients (46%), occurring after a median follow-up time of 25 months (range 2.5–73.1 months). Only 10% of local recurrences occurred later than 5 years after RT. Statistical analysis showed that RT in the relapse situation corresponded to inferior LC rates compared to the primary situation, while other factors such as the GTV, radiation dose (EQD2) and treatment approach (CIRT alone vs. CIRT combined with photons) were insignificant. For OS after RT, patient age and PTV size proved to be significant predictors.

The incidence of late toxicity ≥ III° according to CTCAE v5.0 was 21%. Sacral insufficiency fractures occurred in 49% of patients (maximum III°: 16%) and were thus by far the most frequent late side effect in our analysis. Radiogenic damage to the peripheral nerves, intestinal tract and skin was observed in only 9% (≥ III°: 5%), 3% (all II°) and 9% (all I°) of patients.

**Conclusion:**

Our analysis showed only moderate long-term LC rates after carbon ion-based RT, with sacral chordomas having a particularly poor prognosis in the recurrent situation. Therefore, future studies should evaluate the safety and effectiveness of further dose escalation and hypofractionation of RT in sacral chordoma and weight potential benefits of dose escalation against side effects.

## Background

Chordomas are rare primary osseous malignant tumors that arise along the craniospinal axis, mainly in the skull base and sacrum [1]. The treatment of sacral chordoma is a multidisciplinary task and, despite all the progress made in recent years, remains a great challenge. Because the symptoms are initially only minor, early diagnosis is very rare, so that the tumors are often very large and characterized by locally aggressive growth when they are diagnosed. Surgical resection is regarded as a mainstay of therapy. However, as sole treatment modality it is associated with relatively high locoregional recurrence rates, whereby the size of the tumors, incomplete tumor resections and tumor recurrences seem to have a negative effect on local control (LC) [2–5]. Pathologically complete resections can only be achieved in about 50% of the cases, although the question of the required safety margins is still subject to discussion [5–7]. Therefore, postoperative radiotherapy (RT) is generally recommended to improve LC, even after pathologically complete tumor resections [8]. A further major problem after sacrectomy is the resulting large bone soft tissue defect, which is associated with considerable morbidity and often requires costly surgical measures to cover the defect. High sacrectomies (above the level S2/3) carry the highest risk for postoperative side effects including protracted wound healing disorders, restricted mobility and complete paralysis of the bladder and rectum [8].

Due to the risks of surgery, some patients reject surgical procedures. Other patients experience inoperable recurrences or are not operable because of their comorbidities. For all those patients, definitive RT with particles is an alternative and at least an equivalent option to primary surgical resection with less side effects [4].

However, only a few long-term study data are available regarding response and toxicity after particle-based irradiation of sacral chordoma [4, 9].

Therefore, we have updated the data of a previously published patient cohort in order to assess long-term tumor response and late toxicities after high-dose, heavy ion-based RT [10].

## Patients and methods

### Patients

This retrospective analysis comprised 68 patients with histologically proven sacrococcygeal chordoma who received high-dose carbon-ion based RT at the Heidelberg ion therapy facility in the time period between November 2009 and December 2013. The independent ethics committee of the Heidelberg University Medical Faculty approved this retrospective study (S-165/2012).

### Treatment

**Fig. 1:**
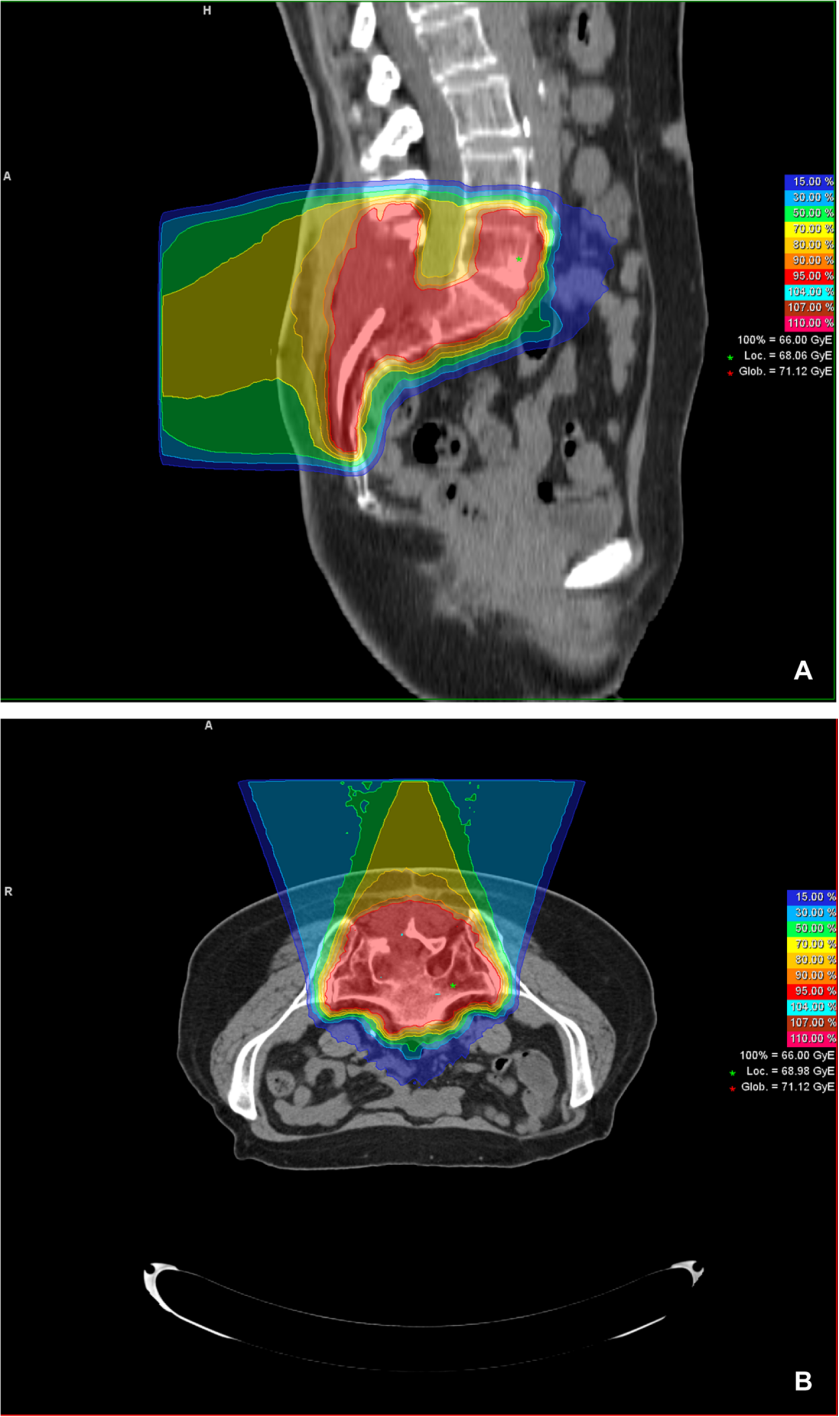
Typical dose distribution of a carbon ion plan with 66 Gy (RBE) total dose (**a**: Sagittal plane, **b** Axial plane)

Patients were assigned to radiation treatment either with carbon ions alone or combined photons and carbon ions after treatment planning with CT and pelvic MRI scans. The preferred MRI sequences for registration with the planning CT’s were transverse STIR and contrast-enhanced T1 sequences. The majority of patients (*n* = 52; 77%) received a primary treatment, whilst the remaining patients were treated in the recurrence situation. None of the patients with a tumor relapse had received RT before. In 40 patients (59%), a surgical resection of macroscopic tumor was performed prior to radiation. A macroscopic tumor was present in 54 patients (79%) at the beginning of irradiation, while the remaining patients (*n* = 14; 21%) had a R0 or R1 situation after surgery. Target volumes and organs at risk were contoured using the Siemens Oncologist software tools (Siemens, Erlangen, Germany). Macroscopic tumor volume based on MRI was defined as the gross tumor volume (GTV). For the primary plan, the clinical target volume (CTV2) comprised not only the GTV or tumor bed but also an extended safety margin usually including the whole sacrum to account for the typical ways of locoregional tumor dissemination. In more than half of the patients (*n* = 40; 59%) a shrinking field technique was used to deliver a radiation boost; therefore, the target volume was reduced to the GTV and/or the tumor resection cavity with an additional small safety margin of 3–5 mm (CTV1). Furthermore, the clinical target volumes (CTV1 and CTV2) were expanded by 3–7 mm to generate the planning target volumes (PTV1 and PTV2). The study patients were either treated with carbon ions alone (*n* = 46; 68%) or bimodal radiation treatment (*n* = 22; 32%) including a combination of photons (primary plan) and carbon ions (boost plan) (see Fig. 1). The equivalent dose to 2 Gy single dose fractions (EQD2) to compare the different fractionation regimes was calculated with a tumor alpha/beta of 2 according to the local effectiveness model 1 (LEM 1) [11]. The median dose administered was 80 Gy with a range of 68.8 to 96 Gy (EQD2). The main risk organs were the intestinal and neuronal structures. For the rectum a D2 < 70 Gy RBE and a dose of less than 30 Gy RBE in the anterior third of the rectum and for the small intestine a D2 < 50 Gy RBE were used as EQD2 related constraints. In contrast, neuronal structures were not spared, since the chordomas of our patient cohort mostly infiltrated the foramina sacralia and therefore these were included in the GTV. Furthermore, the differentiation of the neuronal structures from the tumor tissue is in principle very difficult.

To ensure precise administration of the irradiation, a daily IGRT was performed either with X-rays in 2 planes (carbon ions) or CBCT (photons).

### Follow up examinations

The follow up examinations included regular MRI scans of the pelvis, which were performed at 3-monthly intervals in the first year after RT and at 6-monthly intervals in the following years. For this analysis the follow-up was carried out until the 1st of January 2018. Local progression was defined as an increasing maximum tumor diameter of at least 10% or development of new tumor formations within the irradiated region based on analysis of MR images by a board-certified radiologist (TB). LC, progression-free survival (PFS), metastasis-free survival (MFS) and overall survival (OS) were assessed. Late toxicities were captured retrospectively from the patients’ charts and were defined as progression of symptoms without radiological or clinical signs of tumor progression.

### Statistics

Statistical analysis was performed with the R environment of statistical computing (version 3.5.1, R Core Team 2018, Vienna, Austria). A *p*-value of *p* < .05 was considered statistically significant. The survival time after RT was plotted according to the Kaplan-Meier method. Log-rank testing was applied to analyze univariate group differences in the survival after RT. Furthermore, multiple continuous potentially prognostic factors of survival and local tumor control were evaluated using univariate and multivariate Cox regression models.

## Results

### Follow-up

The median follow-up time after radiation treatment was 60.3 months (range 1.3–97.4 months). Overall, 72% of the patients were followed-up for at least 5 years or until death. The lost to follow-up rate after 1, 2 and 5 years amounted to 3% (2 patients), 7% (5 patients) and 13% (9 patients), respectively. The baseline characteristics of the study population are listed in Table 1.

**Table 1:** Patients’ properties

Characteristics		Value	%
Age (years)			
Median		61	
Range		34–84	
Gender (n)			
Female		22	32.4
Male		46	67.6
Resection status (n)			
Biopsy		28	41.2
R2		26	38.2
R0/1		14	20.6
Treatment (n)			
Primary		52	76.5
Recurrent		16	23.5
Most cranial level of tumor (n)			
L4/5		7	10.3
S1		12	17.7
S2		23	33.8
S3		14	20.6
S4		5	7.4
S5		4	5.9
Os coccygeum		3	4.4
GTV (ml)			
Median		182	
Range		0–1727	
CTV1 (ml)			
Median		263	
Range		0–1743	
CTV2 (ml)			
Median		938	
Range		60–2577	
PTV1 (ml)			
Median		414	
Range		0–2325	
PTV2 (ml)			
Median		1109	
Range		84–3138	
Radiation dose	ED_2Gy_		
Carbon ion only:	(α/β = 2)		
60 Gy/3 Gy (RBE)	75.0 Gy	16	23.5
63 Gy/3 Gy (RBE)	78.8 Gy	2	2.9
66 Gy/3 Gy (RBE)	82.5 Gy	14	20.6
64 Gy/4 Gy (RBE)	96.0 Gy	14	20.6
IMRT + carbon ion boost			
50 Gy/2 Gy + 15 Gy/3 Gy (RBE)	68.8 Gy	1	1.5
50 Gy/2 Gy + 18 Gy/3 Gy (RBE)	74.5 Gy	1	1.5
50 Gy/2 Gy + 24 Gy/3 Gy (RBE)	80.0 Gy	20	29.4

*Abbreviations*: *GTV* Gross tumor volume, *CTV1* Clinical target volume (Boost plan), *CTV2* Clinical target volume (primary plan), *RBE* Relative biological effectiveness, *IMRT* Intensity-modulated radiotherapy

### Local control

Local recurrence was observed in 31 patients (46%), occurring after a median follow-up time of 25.3 months (range 2.5–73.1 months). Most local recurrences were unifocal and occurred in the PTV2 region (24/31 cases, 77%); the remaining recurrences were either multifocal and originated in the PTV1 and PTV2 region (3/31 cases, 10%) or unifocal but separated from the primary tumor in the PTV1 region (4/31 cases, 13%). The majority of local relapses (22/31 patients; 71%) occurred within the first 3 years after primary treatment, but in 3 patients (10%) local relapse was observed later than 5 years after irradiation. For the overall study population, the 1-, 2-, 3- and 5-year probabilities for local tumor control were 90% (95% CI 81–95%), 80% (95% CI 70–89%), 65% (95% CI 54–77%) and 53% (40–67%), respectively.

**Fig. 2:**
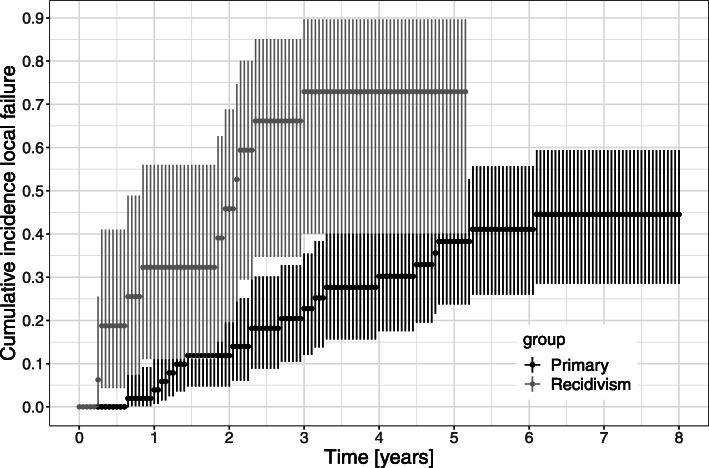
Cumulative incidence of local failure was estimated using the Kaplan - Meier method. Comparison was made between patients with a primary disease situation and recurrent disease situation

The majority of patients with local relapses received systemic therapy, re-irradiation and/or surgical resection; only 4 patients decided for a wait-and-see approach. Amongst the tested prognostic factors, only the therapy situation before irradiation (primary vs. recurrent tumor) proved to be statistically significant for prediction of local tumor relapse (see Table 2 and Fig. 2). For patients with a primary disease, local tumor control after 1, 2, 3 and 5 years was 96% (95% CI 88–99%), 88% (95% CI 78–95%), 77% (95% CI 65–88%) and 62% (95% CI 47–76%). In patients with recurrent chordomas, the 1-, 2-, 3- and 5-year LC rates reached only 68% (95% CI 44–89%), 54% (95% CI 31–80%), 27% (95% CI 10–60%) and 27% (95% CI 10–60%), respectively.

**Table 2:** Analysis of prognostic factors related to local control after RT

	Univariate testing			Multivariate testing		
Parameter	***p***-value	HR	95% CI	***p***-value	HR	95% CI
EQD2 (Gy)	0.13	0.95	0.90–1.01	0.14	0.96	0.90–1.02
Therapy situation (primary vs. recurrent tumor)	< 0.001	4.25	1.99–9.09	< 0.001	4.55	2.06–10.08
GTV (ml)	0.59	1.21	0.60–2.46	0.16	1.70	0.81–3.57
Treatment approach (C12 vs. C12 + Ph)	0.49	1.29	0.63–2.64	NA	NA	NA

*Abbreviations*: *HR* Hazard ratio, *95% CI* 95% confidence interval, *EQD2* Cumulative equivalent radiation dose, *GTV* Gross tumor volume, *C12* Carbon ions, *Ph* Photons, *NA* Not analyzed

In contrast, the GTV, treatment approach (carbon ions only vs. combined photons and carbon ions) and applied cumulative equivalent radiation dose (EQD2) were statistically insignificant for LC in the Cox regression analysis (see Table 2).

### Distant metastases

Six patients (9%) developed distant metastasis, half of which were located in the pelvis. Extra-pelvic metastases manifested in the autochthonous back muscles of the thoracic and lumbar spine (2 patients) and in the lungs (1 patient). In all patients, local relapse was diagnosed prior to (2 patients) or concomitant to metastatic spread (4 patients). The median time until first distant failure of the disease was 32.5 months (range 12–67 months) after carbon-ion based RT. Distant metastases were treated with surgical resection and/or systemic therapy (e.g. tyrosin kinase inhibitors). The 1-, 2-, 3- and 5-year MFS rates were 91% (95% CI 64–100%), 81% (95% CI 54–97%), 71% (95% CI 45–93%) and 52% (95% CI 30–79%), respectively.

### Survival

**Fig. 3:**
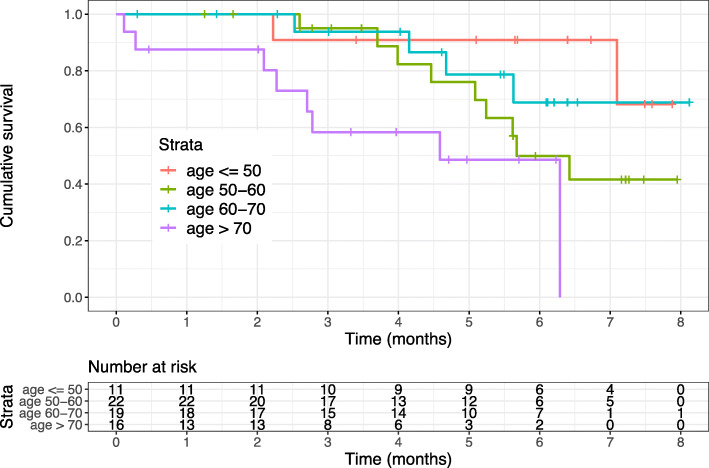
After stratification of patients into 4 age groups a comparison was made between them regarding the overall survival (*p* = 0.01, log-rank test)

**Fig. 4:**
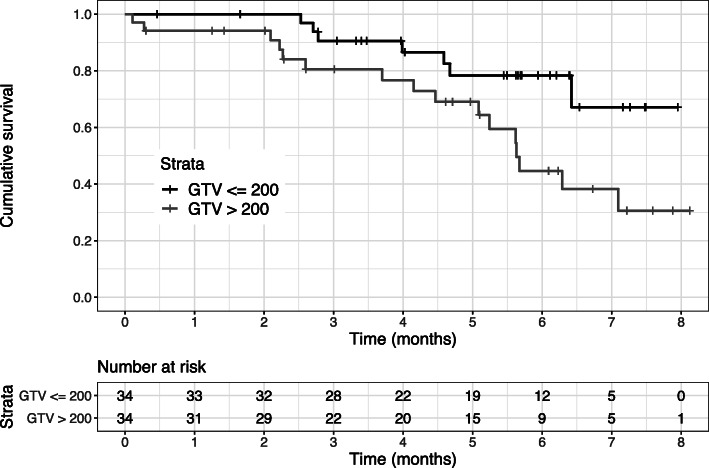
Overall survival (OS) was estimated using the Kaplan - Meier method. Comparison was made between patients with a gross tumor volume (GTV) of > 200 ml vs. <= 200 ml (*p* = 0.003, log-rank test)

**Fig. 5:**
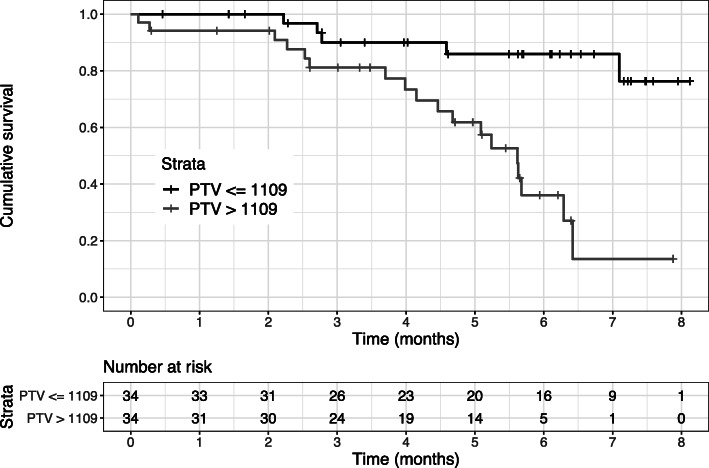
Overall survival (OS) was estimated using the Kaplan - Meier method. Comparison was made between patients with a planning target volume (PTV) of > 1109 ml vs. <= 1109 ml (*p* = 0.0001, log-rank test)

Twenty-three patients (34%) died during follow-up. In 17 of those 23 patients (74%), local relapse with or without additional distant metastases have been diagnosed before death. After a follow-up period of 1, 2, 3 and 5 years, the PFS rates were 90% (95% CI 81–95%), 80% (95% CI 70–89%), 65% (95% CI 54–77%) and 53% (40–67%), while the OS rates amounted to 97% (95% CI 93–100%), 97% (95% CI 93–100%), 86% (95% CI 77–95%), and 74% (95% CI 63–86%), respectively. Univariate log-rank tests found statistically significant associations of the patient’s age at diagnosis group, GTV group and the PTV2 (primary plan) group with OS after RT of sacral chordomas (*p* = 0.01, *p* = 0.02 and *p* = 0.0001), see Figs. 3, 4 and 5. Further tested factors including the patients’ gender, the radiation dose, the localization of the tumor, the CTV1 (boost plan), CTV2 (primary plan), PTV1 (boost plan), disease situation (primary vs. relapsed disease) or the radiooncological therapy approach (carbon ion irradiation alone or combined irradiation with photons and carbon ions) were not statistically significant for survival prediction after RT in the log-rank test.

In univariate Cox models for continuous predictors, patients’ age at the beginning of RT, PTV1 and PTV2 were found to have statistically significant associations with OS after RT (see Table 3). In the multivariate analysis, none of the factors tested showed statistical significance for prediction of OS (see Table 3).

**Table 3:** Analysis of prognostic factors related to overall survival after RT

	Univariate testing			Multivariate testing		
Parameter	***p***-value	HR	95% CI	***p***-value	HR	95% CI
Patients’ age (y)	0.01	1.055	1.013–1.10	NA	NA	NA
EQD2 (Gy)	0.48	0.971	0.895–1.05	0.533	0.975	0.899–1.06
Level of proximal invasion ≥ S2	0.21	1.765	0.725–4.30	NA	NA	NA
Recurrent tumor	0.31	1.593	0.654–3.88	0.36	1.539	0.628–3.77
GTV (ml)	0.06	1.001	1.00–1.00	NA	NA	NA
PTV1 (ml)	0.04	1.001	1.00–1.00	NA	NA	NA
PTV2 (ml)	0.002	1.001	1.00–1.00	NA	NA	NA

*Abbreviations*: *HR* Hazard ratio, *95% CI* 95% confidence interval, *EQD2* Cumulative equivalent radiation dose, *GTV* Gross tumor volume, *PTV1* Planning target volume (boost plan), *PTV2* Planning target volume (primary plan), *NA* Not analyzed

### Toxicity

At baseline, most patients had tumor- or operation-related complaints, with pain, sensitivity and bladder emptying disorders being the most common impairments; other initial complaints were motor deficits, rectal disorders and urinary and fecal incontinence (see Table 4). The operated patients had considerably more neurological impairments than the patients without surgery (bladder emptying disorders: 48% vs. 29%, rectal disorders: 23% vs. 18%, urinary and fecal incontinence: 18% vs. 11%, sensitivity disorders: 35% vs. 21% and motor deficits: 8% vs. 0%). Furthermore, 6 patients had an enterostoma, all affected patients had a sacral chordoma at level S1–3 and 5 patients had a previous surgery.

**Table 4:** Late morbidities (CTCAE v5.0) after carbon-ion based radiotherapy for primary and recurrent sacral chordoma

Grade	0	1	2	3	4
Skin	62	6	0	0	0
Gastrointestinal tract	66	0	2	0	0
Peripheral nerves	62	1	2	1	2
Sacral insufficiency fractures	35	21	1	11	0

The values given are patient numbers

*Abbreviations*: *EQD2* Cumulative equivalent radiation dose, *GTV* Gross tumor volume

After RT, 40 patients (59%) developed radiogenic late toxicities, which affected the bone and nerve tissues, the gastrointestinal tract and the skin (see Table 4). The incidence of late toxicities ≥ grade 3 was 21% (14 of 68 patients). Of the 14 patients with severe radiogenic late toxicities, 12 received a radiation dose of at least 80 Gy (EQD2). Sacral insufficiency fractures (SIFs) were by far the most common late side effect in our analysis accounting for 49% of the patients (33 of 68 patients). Of these, 36% (12 of 33 patients) were symptomatic with considerable impairments in everyday life and severe pain requiring multiple pain medications as well as intensive care by a pain specialist. The median time until diagnosis of SIFs was 12 months (range 1–62 months), whereby the majority of SIFs occurred within the first 2 years after RT (85%, 28 of 33 patients).

Radiogenic damage to the peripheral nerves, intestinal tract and skin was much less frequently observed with an incidence of 9% (6 patients), 3% (2 patients) and 9% (6 patients), respectively (see Table 4).

## Discussion

In our analysis, 46% of the study patients experienced a local relapse after a median follow-up period of 25 months (range 2.5–73.1 months). The majority of local relapses (90%) occurred in the first 5 years after primary therapy including heavy ion-based irradiation, resulting in a 5-year LC rate of 53%, which is in line with other retrospective studies [6, 12, 13]. In contrast to the frequent local recurrences, the distant metastasis rate in our analysis was relatively low (9%) compared to other retrospective studies (range 19–40%) [4, 14–16].

In our analysis, 94% of patients suffered from pain and/or neurological deficits prior to RT. The most serious impairments were observed in patients who had previously had a sacrectomy, including chronic pain, bladder/rectum disorders and urinary and fecal incontinence. In contrast, the tolerability of the radiation treatment in our study was relatively good. Serious side effects occurred in a total of 21% of the study patients affecting the nerve and bone tissues, with most affected patients (86%) receiving a dose of at least 80 Gy (EQD2). None of the study patients developed urinary or fecal incontinence, severe gastrointestinal toxicity or permanent severe skin damage. By far the most common late toxicity were SIFs, which occurred in half of our study patients (49%). However, only one third of these fractures (36%) were clinically symptomatic, with severe pain being the main complaint. To this date, there are only 2 other retrospective studies that have specifically investigated the occurrence of sacral fractures after high-dose irradiation of chordomas and reported similar fracture rates [17, 18]. In our analysis, most fractures (85%) occurred within the first 2 years after RT.

During follow-up, 34% of our study patients died. Of these, 74% had disease progression prior to death. For the entire study population, the 5-year LC, PFS, MFS and OS rates were 53, 53, 52 and 74%, respectively. In our analysis, RT in the relapse situation was found to correspond to inferior LC rates compared to the primary situation, which is consistent with the results of a study by the Massachusetts General Hospital (MGH) [19]. Since complete tumor resections are often not possible due to the topographic proximity to critical structures and a large number of local recurrences could not be prevented in our analysis, a preoperative RT strategy is a reasonable alternative option. However, sacral chordomas require high radiation doses and the administration of the entire dose preoperatively carries a high risk of chronic postoperative wound healing disorders.

In summary, the main challenge in the treatment of sacral chordoma is to achieve permanent local control after primary treatment. Compared to our results, other particle centers reported better LC rates. The MGH, for example, reported 5-year LC rates of 60% after high-dose, proton-based irradiation with or without surgery after a median follow-up of 41 months [3]. The Nationale Institute of Radiological Sciences (NIRS) in Chiba, Japan could show even better results than the MGH with 5-year LC rates of 77% [4]; in this study, patients received primary hypofractionated heavy ion therapy with a median dose of 70,4 Gy in 16 fractions over 4 weeks. The median follow-up period was 62 months. In total, 29% of local recurrences occurred later than 5 years after irradiation.

However, the comparison of previous study results is complicated by the differences in radiation modalities, doses and fractionation, biological models, the populations studied (primary vs. relapse situation, definitive vs. postoperative situation), tumor volumes and target volume definitions, follow-up periods and local relapse definition. In our study 79% of the patients had a macroscopic tumor (median volume 243 ml, range 5–1727 ml) and 23% of the patients were irradiated in the local relapse situation. Therefore, our study population represents rather an unfavourable collective compared to many other retrospective studies [4, 20, 21].

Due to the fact that chordomas grow slowly, long follow-up periods are necessary; local recurrences often occur later than 5 years after primary therapy and even after 10 years of follow-up no plateau of local recurrence rates is reached [8]. Therefore, when comparing studies, the follow-up time must be taken into account.

A further problem is that there is no uniform definition of local relapse. In many studies there is no detailed information on this or reference is made only to the radiologist’s assessment [3, 4, 21]; Chen et al. defined the local recurrence as an increase in size of chordomas in 2 consecutive follow-up examinations [20]. In another study the modified Response Evaluation Criteria in Solid Tumors (RECIST) and volumetric analysis were used to evaluate the tumor response to RT [9].

Another critical point is the definition of the clinical target volume, which differed significantly between our study and other retrospective studies. In our study the CTV of the basic plan usually included the entire sacrum and in the boost plan this was reduced to a small GTV-CTV safety margin of 3–5 mm. This field shrinkage technique was also used in the MGH studies [3, 19]. In contrast, In the NIRS and HIMBC studies only one CTV was generated for the entire irradiation series by adding a small safety margin to the GTV (as it was done in our study for the boost plan), i.e. often the entire sacrum was not included in the target volume [4, 21]. Consequently, in many other studies sacral tumor recurrences could only have been classified as locoregional recurrences if they were out-of-field or at the edge of the field, whereas in our study they were always classified as local recurrences. Therefore, PFS may be a better comparison parameter than LC when comparing the results of different retrospective series. For instance, LC in the NIRS patient collective was distinctly higher than in our collective (5-year LC 77% vs. 53%), while PFS and OS rates were similar (5-year PFS 50% vs. 53%, 5-year OS 81% vs. 74%) [4]. In this study, univariate analysis found that the patient’s age and the PTV were strong prognostic factors for the OS.

A further problem are the different models in Europe and Japan for prescribing the dose of carbon ion therapy, which makes it difficult to compare the doses applied in the various retrospective studies [22]. Therefore, another possible explanation for the better LC rates of the NIRS study could be the higher cumulative equivalent radiation dose applied (64.0–73.6 Gy RBE). However, the radiation dose was no significant predictor for LC in our analysis.

Our study has several limitations. First, the data set was collected retrospectively. The main problem for conducting prospective studies in sacral chordoma is the rarity of the disease, which is why there are only retrospective studies on this topic so far. Second, the number of patients in our study is limited, which is also explained by the rarity of the disease; however, there are only a few studies with more patients than in our study [2–4]. Third, the median follow-up time of about 5 years is sufficient for many tumors to collect long-term oncological data, but it is not sufficient for sacral chordomas, as these grow slowly and local recurrences therefore often occur later than 5 years after primary therapy [2]. Therefore, we intend to further observe this patient collective and to republish the collected data again. Fourth, the lost to follow-up rate was 13% after 5 years, which is a limitation for the analyzed endpoints of this study (recurrence, toxicity and survival rates). In addition, a randomized phase 2 study is currently underway at the Heidelberg Ion Therapy Center (ISAC), which compares hypofractionated proton and heavy ion therapy in sacral chordoma patients with a dose of 64 Gy RBE in 16 fractions in both therapy arms; the results are still pending [23].

## Conclusion

Our analysis showed only moderate long-term LC rates after carbon-ion based RT, whereby sacral chordomas have a particularly poor prognosis in the recurrence situation. Future studies should take further dose escalation into account to improve LC and PFS. However, possible benefits of dose escalation must be weighed against the risks of treatment. In our study, 21% of the patients experienced severe late toxicities with SIFs being the main clinical problem.

## Data Availability

The datasets used and/or analyzed during the current study are available from the corresponding author on reasonable request.

## Abbreviations

LC: Local control
RT: Radiotherapy
CT: Computed tomography
MRI: Magnetic resonance imaging
GTV: Gross tumor volume
CTV: Clinical target volume
PTV: Planning target volume
PFS: Progression-free survival
MFS: Metastasis-free survival
OS: Overall survival
SIF(s): Sacral insufficiency fractures (SIFs)
MGH: Massachusetts General Hospital
NIRS: Nationale Institute of Radiological Sciences
RBE: Relative biological effectiveness
